# A Review of the Efficacy and Mechanisms of Blood Flow Restriction Training in Enhancing Somatic Function and Preventing Falls in Older Adults

**DOI:** 10.7759/cureus.66375

**Published:** 2024-08-07

**Authors:** Liang Han, Xiaoming Xi, He Wang, Mengfan Kan, Shaohong Yu

**Affiliations:** 1 Rehabilitation Medicine, Shandong University of Traditional Chinese Medicine, Jinan, CHN; 2 Physical Medicine and Rehabilitation, Rehabilitation Center, Beijing Rehabilitation Hospital Affiliated to Capital Medical University, Beijing, CHN; 3 Physical Medicine and Rehabilitation, The Second Affiliated Hospital of Shandong University of Traditional Chinese Medicine, Shandong, CHN

**Keywords:** research progress, falls, prevention, older adults, low-intensity blood flow restriction training

## Abstract

Falls have become an important public health problem that seriously affects the quality of survival of older adults and are a major cause of fractures, death, and reduced quality of life. With the advent of an aging society, the social, economic, and medical burdens of falls in older adults are increasing. Currently, there is a lack of effective means to prevent falls in older adults, and traditional health education and clinical interventions are not effective. It is urgent to find a safe and effective training method that can improve balance function and is suitable for the elderly. Low-intensity blood flow restriction training (BFRT) is an emerging training modality that, by restricting blood flow to the limbs and combining it with low-intensity exercise, can effectively improve muscle mass, aerobic capacity, and bone density, and has been shown to enhance somatic function in older adults. However, the effectiveness and specific mechanisms of BFRT in preventing falls in older adults are unclear. Based on recent research progress, this paper explores the possibility of BFRT in preventing falls in older adults by analyzing its positive effects on muscle mass, balance function, and cognitive function, the risk factors of falling in the elderly are summarized, as well as its potential role in reducing fall risk factors. It aims to provide new thinking for academia and clinical practice and to provide a scientific basis for reducing the risk of falls in the elderly.

## Introduction and background

Population aging is a major challenge facing the world, and the aging situation in China is particularly severe. Data from the seventh national census show that the proportion of the elderly population aged 60 years and above in China has reached 18.7%, and the proportion of the elderly population aged 65 years and above has reached 13.5% [1], and the degree of aging is deepening. The United Nations predicts that the global elderly population will reach two billion by 2050, and the social, economic, and medical burdens of aging will become increasingly heavy [2].

Falls, as a common and serious health problem among the elderly, have become an important issue in public health. In the United States, three older adults die every hour due to falls, and this number is expected to rise to seven by 2030, and this trend is not affected by racial differences [3,4]. Falls in older adults not only lead to physical injuries such as fractures and soft tissue injuries, but also result in decreased self-care ability, prolonged hospitalization, and increased healthcare costs, which seriously affects the quality of life and healthy lifespan of older adults.

The occurrence of falls is influenced by a variety of factors, including both intrinsic factors such as age, gender, functional status, disease conditions, and medications, and extrinsic factors such as environmental factors. The influence of intrinsic factors on falls gradually increases with age [5]. Currently, some progress has been made in interventions aimed at preventing falls in older adults, but the effectiveness still needs to be improved.

Exercise intervention is one of the effective means to prevent falls in older adults. Resistance exercise is considered to be an effective method for fall prevention because it can effectively improve muscle volume and mass in older adults [6]. However, high-intensity resistance exercise poses safety risks for older adults, which may increase the body burden and the incidence of adverse events. Therefore, it has become a research hotspot to find a training method that can enhance muscle strength while avoiding the risk of high-intensity exercise.

Blood flow restriction training (BFRT), also known as KAATSU training, is a training modality that has attracted much attention in recent years. BFR restricts blood flow by applying external pressure proximally to the muscle [7] , thereby performing low-intensity training in a distal ischemic environment. Its core mechanism is to enhance muscle strength, endurance, and cardiorespiratory fitness through a metabolic stress response [8,9]. BFR training is effective in improving somatic function in older adults and has a high degree of safety, making it a viable alternative for older adults who are unable to perform high-intensity resistance exercises.

In recent years, BFRT has been studied in various fields and its application has been expanded. This study will look at the potential role of BFRT in reducing fall risk factors and investigate the mechanisms and effectiveness of BFR in preventing falls in older adults. This study aims to provide new thinking for academia and clinical practice and to provide a scientific basis for reducing the risk of falls in older adults and promoting healthy aging.

## Review

Improvement of somatic function in older adults by BFRT

Muscle Mass

Skeletal muscle mass decreases with age by 3%-8% per decade after the age of 30 years [10], which can have a significant impact on body function and metabolism, including an increased risk of falls and death [11]. Since older adults tend to have comorbidities such as diabetes, coronary artery disease, and musculoskeletal injuries, high-intensity resistance training does not apply to them, whereas BFRT promotes muscular adaptations in diverse populations. However, to date, its effects and mechanisms on muscle mass in the elderly are still understudied. Bigdeli et al. [12] explored the effects of six weeks of BFRT on muscle mass indexes in elderly men through a randomized controlled trial, and the results showed that the BFRT combined functional training group compared with the functional training group alone, and the BFRT combined group reduced the serum C-terminal fragment of collectin (C-terminal agrin-fragment (CAF)) level was more obvious, in addition, the functional training group could significantly reduce the level of procollagen type III N-terminal peptide (P3NP), but there was no significant change in the BFRT combined group. It should be noted that CAF and P3NP are closely related to the neuromuscular junction, so they can be used to assess skeletal muscle mass, with CAF negatively correlated with muscle mass and P3NP positively correlated with muscle mass. The mechanisms involved are that BFRT induces muscle contraction, leading to ischemia and excessive accumulation of metabolites in the muscle fibers and that these metabolites can stimulate afferent nerve III and afferent nerve IV muscle fibers, which leads to the recruitment of fast-twitch muscle fibers, and that fast-twitch muscle fibers are more sensitive to atrophy with age, which may be a mechanism for the greater ameliorative effect of the combined BFRT group. Because aging also has a marked effect on these fiber types, BFRT may be an effective method of stimulating fast-twitch muscle fibers that could be used to attenuate atrophy associated with aging. However, Fragala et al. [13] came up with the opposite result, finding that after six weeks of BFRT, CAF increased in both older men and women. Although the mechanisms responsible for these differences are unclear, several methodological differences could explain these conflicting results. The training intensity in the Bigdeli study was higher than that in Fragala's study (intensity is an important training variable that can influence CAF response to exercise); furthermore, the training protocol in Fragala's study consisted of monoarticular exercises, whereas whole-body functional training was used in the Bigdeli study; furthermore, the participants in the Fragala study were primarily older women, whereas the participants in the Bigdeli study were healthy older men. These several differences may account for the different results. It also just goes to show that muscle mass is a complex indicator that is influenced by a variety of factors such as age, gender, and exercise program and intensity. Neither study measured muscle volume, muscle strength, or muscle composition, which is a key reason for the controversial results. Currently, most studies have concluded that BFRT increases muscle mass and strength [14] by mechanisms that include regulating hormone secretion and promoting the synthesis of proteins and ribosomal organisms. Future studies could explore the effects of different intensities of BFRT on blood markers, functional capacity, and hormonal adaptation in the elderly population and analyze the mechanism of action in depth.

Aerobic Capacity

Maximal oxygen consumption (VO2max) is the gold standard for evaluating aerobic capacity, which represents the amount of oxygen the body can take up at maximum exercise intensity. Beginning at age 25, VO2max decreases by approximately 1% per decade, and by about age 70, VO2max decreases by approximately 20%-30% [15], often accompanied by decreases in cardiorespiratory, muscle mass, circulation, and neurological function, increasing the risk of falls, incapacitation, and death. If VO2max is below the threshold (18 mL/(kg x min) for men and 15 mL/(kg x min) for women), total disability may occur at the age of 80-85 years, and if regular aerobic exercise slows down or reverses the functional deterioration, reduces an individual's physiological age by at least 10 years, and potentially delays the duration of disability [15], so keeping up with aerobic exercise in the elderly is extremely necessary. High-intensity aerobic exercise may have safety risks for older adults, but low-intensity BFRT, as an emerging training modality, is theorized to be effective in improving aerobic capacity as well as cardiorespiratory fitness in older adults, as well as decreasing safety risks, providing a safe and effective aerobic exercise option for older adults. However, with the current research, this theoretical assumption is still controversial. Kun et al. [16] did a meta-analysis on whether BFRT can improve VO2max in the elderly and found that there was no statistical difference in the change of peak VO2max before and after training (P=0.21) and that BFRT could not improve VO2max and cardiopulmonary function. However, this study included only three papers, and the credibility of the results is greatly reduced. However, another study [17] suggested that BFRT is effective in increasing aerobic capacity and improves exercise tolerance and intramuscular adaptations to support endurance exercise, by mechanisms that include increased stroke volume and cardiac output, increased muscle capillary and diffuse oxygen transport, and increased muscle oxidase activity. Park [18], Abe [19], Kancin [18], Christiansen [20,21], and others have also concluded that BFRT improves aerobic capacity. Future studies should expand the sample size and include more detailed evaluation indicators to provide stronger evidence that BFRT improves aerobic capacity in older adults.

Balance and Walking Function

With aging, the body's balance ability will gradually decline, making it more likely to fall. Older people usually have decreased balance control ability and slower reaction speed when standing, walking, turning, and other movements, and are more likely to lose balance. And walking speed gradually slows down. Slower gait speed is an important indicator of aging and is closely related to fall risk and reduced quality of life. In addition, gait changes, including shortened stride length, unsteady stride length, and shuffling stride length, are also observed in the elderly, which are related to factors such as decreased muscle strength, decreased range of motion of joints, and decreased balance function. Liu [22] found in a 12-week randomized controlled trial that, compared with the control group, the BFRT group showed significant improvement in extensor and flexor strength at both angular velocities of the hip, knee, and ankle joints; it also significantly improved the amplitude and velocity of the offset of two-legged standing with eyes open, two-legged standing with eyes closed, one-legged standing with eyes open, and one-legged standing with eyes closed; and significantly improved the Berg Balance Scale Score and the Functional Forward Reach Distance; in addition, it also significantly improved the Get-Up Timed Walk, the 30-second Sit-Stand, and the 10-meter Timed Walk. This study concluded that BFRT can significantly improve balance function and walking ability in older adults. Two other studies [23, 24] also found that BFRT had a positive effect on balance function improvement in older adults. In addition, Yokokawa [23], Bryk [25], and others found that after BFRT, 10-meter walk, rise-timed walk test, maximal stride length, and jump reaction time were significantly improved in elderly subjects. Currently, the efficacy of BFRT to improve balance function and walking ability in older adults is definite, and the mechanisms [22] are mainly related to metabolic stress, mechanical tension, hormonal responses (growth hormone (GH), insulin-like growth factor-1 (IGF-1), testosterone), muscle fiber recruitment, muscle swelling, reactive oxygen species production, and heat shock protein transport and aggregation. Future studies should be conducted in depth in terms of mechanism of action, training programs, population characteristics, and combined interventions to promote the application of BFRT in improving balance function and walking ability in the elderly, and thus enhance their independent living ability and quality of life.

Cognitive Function

As we age, the body experiences a gradual decline in cognitive function, including a decrease in cognitive speed, working memory capacity, executive function, vocabulary and verbal fluency, and spatial cognition, which is mainly related to genetic, lifestyle, disease, and psychological factors. As the population is aging, cognitive dysfunction has become a critical public health issue and can increase the risk of falls and fractures, which will place a heavy burden on the state, society, and families [26]. Aerobic and resistance exercise can improve cognitive executive function in older adults and patients with chronic diseases [27]. However, there are fewer studies on the effects of BFRT on cognitive function. Sugimoto et al. [28] found that a 15-minute BFR walk improved post-exercise executive function, and the mechanism may be related to an increase in blood lactate levels. In addition, Dalsgaard et al. [29] also found that low-intensity aerobic exercise coupled with BFR increased post-exercise brain lactate metabolism. Therefore, the potential effect of BFRT on post-exercise executive function improvement may be due to increased circulating lactate levels and brain lactate metabolism. However, Rasmussen et al. [30] reported that an increase in cerebral lactate metabolism was observed when blood lactate levels exceeded 2 mmol, whereas blood lactate levels after BFR walking were only 1.9 mmol. Therefore, factors other than systemic and cerebral lactate metabolism may play a role in the improvement of executive function after BFR walking [31]. The results suggest that the modulation of exercise-induced perceptual responses (e.g., arousal) may play a role in improving executive function. and BFR arousal was higher after walking. Both BFR aerobic and resistance exercises increased norepinephrine in the blood [32], which is a modulator of arousal [33]. Therefore, the enhancement of the norepinephrine-arousal system may be one of the reasons why BFR walking improves executive function. In the future, the differences in the effects of different training parameters and different training modes of BFRT on the improvement of cognitive function should be deeply investigated, and the mechanism of action should be deeply analyzed in terms of cerebral hemodynamics, brain neurotransmitters, neurotrophic factors, neuronal activity, and inhibition of inflammatory response.

Bone Density

Bone density is an important indicator of bone strength, which decreases with age, increasing the risk of osteoporosis, fractures, and falls. Some studies have shown that BFRT improves bone density and has positive benefits on bone formation in middle-aged and elderly people [34]. However, studies by Fakhri [35], Karabulut [36], Kargaran [37], and others showed that there was no significant difference between the effects of the BFRT group and the low-intensity training group on bone metabolism. The effectiveness of BFRT on bone metabolism is still controversial. Therefore, Wang et al. [38] did a Meta-analysis on the effect of BFRT on bone metabolism, which included 12 papers and 378 subjects, and the results showed that compared with the low-intensity training group, the BFRT group had a greater increment in bone-specific alkaline phosphatase (BALP) and a greater increment in bone mineral density (BMD) than the low-intensity training group. The BFRT group produced smaller increases in BALP, similar increases in BMD, and similar decreases in CTX compared with the high-intensity training group. CTX decreased by a similar amount. This suggests that the BFRT group was more effective at improving bone health than the low-intensity training group, but less effective than the high-intensity training group. Since high-intensity training does not apply to older adults, BFRT may be an effective and efficient way to improve bone health in older adults. The potential mechanisms are also multifaceted.BFRT may increase intramedullary pressure and interstitial fluid by increasing vascular restriction, which has been shown to favor bone metabolism [39]. In addition, BFRT may affect osteoclast activity through hypoxia and a small decrease in pH, which activates factors important for the neovascularization of bone tissue (e.g., hypoxia-induced transcription, vascular endothelial growth), which transport osteoblasts and osteoclasts that are essential for bone remodeling [40]. In addition, hormones (e.g., cortisol, testosterone, IGF-1) play an important role in regulating bone metabolism by promoting bone formation or inhibiting bone resorption [41]. Future studies should establish a scientific evaluation index system to assess the improvement effect of BFRT on bone density, bone microstructure, and bone growth factors.

Fall risk factors and the potential role of BFRT

Falls in the elderly are a multifactorial, multilevel, and complex problem that needs to be analyzed and assessed from a variety of physiological, psychological, environmental, and social aspects.

Intrinsic Factors

Physiological factors: Bone health - osteoporosis, low bone density, and fractures lead to fragile bones and make fractures more likely to occur; muscle strength and endurance - decreased muscle strength and endurance in the lower limbs affects balance control and walking stability and makes falls more likely to occur; joint range of motion - decreased range of motion of joints affects gait smoothness and coordination and makes falls more likely to occur; sensory and perceptual functions - decline in perceptual functions such as vision, hearing, proprioception, etc., affects the perception of body posture and movement, leading to a decrease in balance, making it more likely to fall; cardiovascular function - cardiovascular diseases, heart rate disorders, and fluctuations in blood pressure, etc., can lead to dizziness and syncope, increasing the risk of falling; neurological disorders - neurological disorders, such as strokes, Parkinson's disease, cerebral infarction, and cerebral hemorrhage, can lead to balance dysfunction, gait abnormality, increasing the risk of falling.

Drug factors: Some drugs, such as sedatives, antidepressants, antihypertensive drugs, diuretics, etc., can affect balance function, cognitive function, and reaction speed, increasing the risk of falls.

Psychological and mental factors: Factors such as depression, anxiety, and cognitive disorders can affect attention, reaction speed, and judgment, increasing the risk of falls.

Extrinsic Risk Factors

Environmental factors: Home environment - insufficient light in the room, slippery floor, no handrail on the stairs, obstacles on the ground, etc., will increase the risk of falling; outdoor environment - uneven road surface, ice and snow on the ground, traffic chaos at intersections, etc., will increase the risk of falling; wearing - inappropriate shoes, clothes too long or too tight, socks too slippery, etc., will affect the balance and movement and increase the risk of falling.

Social factors: Social support - older people who lack social support, such as help from family, friends, neighbors, etc., are more likely to fall; economic status - older people who may not be able to afford the necessary assistive devices or to undergo the necessary rehabilitation treatments because of their economic status are more likely to fall.

Other Risk Factors

Age, gender, race, and history of falls.

Mechanisms of BFRT to reduce fall risk

This paper analyzes the potential mechanisms of BFRT to prevent falls in older adults, mainly based on intrinsic physiological factors and cognitive functions.

Muscle Mass and Strength Enhancement

BFRT can effectively increase the muscle mass and strength of the elderly, which plays a crucial role in preventing falls in the elderly.

Effect on Balance Control Ability

Strong lower limb muscles, especially quadriceps and gastrocnemius, can effectively improve the body's balance control ability [42]. These muscles are responsible for maintaining the body in an upright position and responding to body tilts with rapid adjustments. As muscle strength increases, the synchronization between motor units (i.e., muscle fibers and the motor neurons that innervate them) increases, resulting in more coordinated and powerful muscle contractions [43]. This increased coordination helps optimize movement patterns, reduces unnecessary energy expenditure, and enhances perception and control of movement states, resulting in improved balance control. It also promotes adaptive changes in the central nervous system that include enhanced connectivity between neurons, increased synaptic plasticity, and modulation of neurotransmitter release [43]. Allowing the CNS to process information from proprioceptors more accurately and respond more quickly to maintain body balance.

Effect on Neuromuscular Reaction Speed

Improvement in muscle mass and strength also improves neuromuscular reaction speed, faster response, timely adjustment of body posture, and avoidance of falls. The specific mechanisms are the improvement of nerve conduction efficiency (including the recruitment of spinal motor neurons and the increase of discharge frequency) [44], the optimization of spinal and corticospinal pathways [45], the change of motor neuron excitability (manifested by the action potential that can be generated at lower thresholds, which accelerates the process of initiating muscle contraction) [46], and the shift in muscle fiber type (which leads to the shift of muscle fiber type from slow muscle fibers to fast muscle fibers or increase the proportion of fast muscle fibers, which have higher contraction speed and force, and therefore can respond to nerve impulses faster) [47], increase in muscle cross-sectional area (which helps the muscle overcome internal and external resistances faster and increase the contraction speed) [48], and increase in the efficiency of muscle contraction (improvement of the contraction mechanism of the muscle, including an increase in the speed of myofilament sliding, increase in the efficiency of calcium release from the sarcoplasmic reticulum, etc.) [49], increase in the efficiency of muscle contraction (improvement of the contraction mechanism of the muscle, including an increase in the speed of myofilament sliding, increase in the efficiency of calcium ion release efficiency) [49] and enhanced neuromuscular coordination (including improved synchronization of motor units and tuning of antagonistic muscle coactivation) [44], among many others. Together, these changes allow the muscles to produce a faster contraction response after receiving a nerve impulse, thus improving overall motor performance and reaction speed.

Effect on Proprioceptive Functions

Improvements in muscle mass and strength can improve proprioceptive function, increase the ability to perceive body position and movement, enhance balance control, and reduce the risk of falling. Muscles are richly distributed with proprioceptive receptors, such as muscle shuttles and Golgi tendon organs, which are responsible for sensing changes in muscle length, tension, and joint angles, and can more accurately sense muscle contraction and relaxation, thus giving information to the brain more efficiently [50].

Effects on Gait

Improvements in muscle mass and strength can improve gait stability. This includes the ability to improve stride length and frequency, making the gait more stable and reducing the risk of falls caused by unstable gait; the ability to improve walking speed, enabling older adults to respond more flexibly to emergencies, making it easier to avoid obstacles promptly and reducing the risk of falls; and the ability to improve gait coordination, increasing the stability of the body during walking and reducing the risk of falls caused by gait abnormalities. It is closely related to several aspects of mechanisms such as balance, coordination, muscle tone balance, sensory function and spatial cognitive function, central control, and improvement of muscle mass, which work together to enable individuals to maintain a stable gait pattern, reduce the risk of falling, and improve the quality of life.

Improved Aerobic Capacity

Although there are different conclusions as to whether BFRT improves aerobic capacity and cardiorespiratory fitness in the elderly, most studies have concluded that there is a positive benefit between the two, and there are studies on the related mechanisms to provide scientific evidence. Increased aerobic capacity can enhance the contraction capacity of the heart and provide cardiac output, so that blood can be transported more efficiently throughout the body, including muscle tissue, which can provide sufficient oxygen and nutrients to the muscles, and improve muscular endurance and function; it can also improve the elasticity of the blood vessels, reduce vascular resistance, promote blood circulation, and increase the blood supply to the muscles, which helps to increase the utilization rate of oxygen in the muscles, and improve the muscle's metabolism and function; in addition, it also helps to reduce resting heart rate and enhance cardiac regulation [51], enabling the heart to better adapt to the demands of exercise and daily activities, and reducing the risk of falls due to decreased cardiovascular function. Increased aerobic capacity can effectively reduce the risk of falls in older adults through a variety of mechanisms, including enhancing cardiorespiratory function, improving bone health, increasing muscle strength and endurance, improving balance and coordination, and improving psychological status.

Cognitive Function Promotion

BFRT can effectively improve cognitive function in older adults, which is crucial for the prevention of falls.

Improvement of Perception of the Environment

Improvement of cognitive function can enhance visual perception ability, such as improving the ability to discriminate color, contrast, depth, etc., which can help older people identify the surrounding environment more accurately and avoid falls due to visual impairment. It also enhances auditory perception, for example, improving the ability to recognize the direction, distance, and type of sound, helping the elderly to more accurately perceive their surroundings and avoid falls due to auditory impairment. In addition, it can enhance spatial cognitive ability, such as improving the ability to judge distance, direction, and location of objects, helping the elderly to better plan their walking routes and avoid falls due to spatial cognitive impairment.

Enhance Judgment and Decision-Making Ability

Improvement in cognitive function can enhance risk assessment ability, helping older people to recognize potential fall risks, such as uneven ground, insufficient light, obstacles, etc., and take timely measures to avoid falls. It also enhances decision-making ability, helping older adults to make more rational judgments, such as choosing appropriate walking routes and avoiding dangerous areas, thus reducing the risk of falls. In addition, it can improve reaction speed and help older people react faster, for example, when they suddenly encounter obstacles or the ground is not smooth, they can make timely adjustments to avoid falling.

Improvement of Executive Functions

Improvement of cognitive function can enhance planning and organizing ability, helping older people to make safe walking routes and arrange action steps, and reduce falls caused by poorly planned plans of action. It can also enhance the ability of attention allocation, help older people to focus their attention, reduce being attracted by the surrounding environment and forgetting the goal, and avoid falls caused by distraction. In addition, it is able to enhance working memory ability and help older adults remember walking routes, keep goals, etc., to reduce falls caused by memory loss.

Enhancement of Emotion Regulation Ability

Improvement of cognitive function can alleviate anxiety and depression, enhance emotion regulation ability [52], help older adults maintain a positive and optimistic state of mind, and reduce mobility disorders and the risk of falls caused by emotional problems. It can also reduce fear and worry, enhance self-confidence, help older adults actively participate in daily life, and reduce activity limitations and fall risks due to fear and worry.

Promote the Improvement of Other Functions and Indirectly Reduce Fall Risk

Improvement in cognitive function can promote social participation, such as participating in social activities and learning new skills, thus improving quality of life and reducing fall risk.

Increase in Bone Density

BFRT can effectively enhance bone density in the elderly, which plays a crucial role in preventing falls. Bones serve as attachment points for muscles and provide support for muscles. Increased bone density can enhance the strength and stability of the bones, providing more stable support for the muscles. It can also enhance the carrying capacity of the bones so that the muscles can withstand greater loads, thus promoting the growth of muscle strength and improving balance function. In addition, the bone releases some signaling substances that affect muscle growth and function, and increased bone density can optimize the transmission of these signals [53], promoting the growth of muscle strength and the improvement of balance function. There are also some proprioceptive receptors in the bones, which can sense the position, movement, and load of the bones [54] and transmit their information to the brain. It can improve the transmission efficiency of skeletal signals and enhance the brain's ability to perceive the state of bones, which in turn improves proprioceptive functions and balance control.

Current status and challenges in the application of BFRT in the prevention of falls in older adults

Current Status of Application

As an innovative training method, BFRT is mainly applied in the field of sports injury and musculoskeletal rehabilitation [55], which has positive effects on muscle mass and strength enhancement, aerobic capacity improvement, cognitive function improvement, bone density increase, and physical mobility improvement. In recent years, it has shown significant application potential and research value in the field of fall prevention in the elderly. In hospitals and community rehabilitation centers, BFRT has been widely used in the rehabilitation training of elderly patients. For example, for elderly people with myasthenia gravis and osteoarthritis who have decreased muscle strength, BFRT can significantly improve their lower limb muscle strength, improve gait stability, and reduce the incidence of falls. Although BFRT performs well in preventing falls, its safety still requires attention. During clinical application, the pressure value of the pressurization device should be strictly controlled to avoid excessive compression leading to limb ischemia or nerve damage. At the same time, personalized training programs should be developed according to the specific conditions of patients to ensure the safety and effectiveness of training. In addition, more and more animal and human experiments have confirmed the biological mechanism of BFRT in preventing falls in the elderly. The core mechanism lies in the stimulation of metabolic stress and the promotion of muscle growth and strength enhancement by restricting the local hypoxic environment caused by blood flow [56]. Specifically, the reduced blood flow during BFRT results in a lower oxygen supply to the limb, and the localized hypoxic stimulus increases the rate of muscle metabolite production while decreasing the muscle's ability to remove metabolites, ultimately leading to the accumulation of intramuscular metabolites. This accumulation stimulates the secretion of GH and IGF-1, which are essential for muscle growth. It also promotes protein synthesis and cell proliferation and differentiation by regulating the PI3K/AKT/mTOR signaling pathway, which further enhances muscle mass and strength [56]. However, further in-depth studies on its biological mechanisms, optimization of training protocols, and improvement of safety and effectiveness are still needed to better serve the health needs of the elderly population in the future. At the same time, strengthening scientific publicity and education is also an important way to promote the fall prevention strategy of BFRT.

Problems and Challenges

Limited research evidence: Current research on BFRT to prevent falls in older adults is limited, and more high-quality clinical studies are needed to further validate its effectiveness and safety. Current studies mainly focus on short-term effects and lack long-term follow-up data to assess its long-term effects and safety fully.

Training specifications have not been fully standardized: there are differences between different studies, and more standardized training programs and criteria need to be developed to ensure the safety and effectiveness of training. For example, there are differences in parameters such as strap pressure, training intensity, and training frequency used in different studies, and more uniform standards need to be developed.

Professional system has not been fully established: Currently, there is a lack of professionals in BFRT in China, which is not enough to ensure the safety and effectiveness of the training. Therefore, it is necessary to strengthen the professional training for rehabilitation physicians, rehabilitation therapists, etc., to improve their mastery and application of BFRT.

Insufficient popularization and promotion: At present, BFRT equipment and professional personnel are not well equipped, which limits its application among the elderly, and the promotion and popularization of BFRT need to be strengthened so that it can be more easily accepted and applied by the elderly. The training of professional staff requires time and money, which limits its promotion and application in primary care organizations and communities.

Individualized differences in the elderly: There are large individual differences in the elderly population, and the training effect of BFRT may vary from person to person, individualized training programs need to be formulated according to individual conditions to ensure the effectiveness and safety of training.

Future research direction and suggestions

In-Depth Exploration of Mechanism Research

Multi-omics research: utilize proteomics, genomics, metabolomics, and other technical means to explore in-depth the mechanism of BFRT's influence on the muscle, bone, nervous system, and other multi-levels of the elderly.

Signaling pathway study: To investigate which signaling pathways are activated by BFRT, such as mTOR pathway, AMPK pathway, etc., to promote muscle growth, enhance vascular function, improve bone metabolism, and improve neuromuscular control ability.

Individualized research: Combining the individual differences of the elderly, such as age, gender, health status, activity ability, etc., to study the mechanism of BFRT's action on different individuals, and to formulate a more personalized training program.

Further Validation of Effectiveness Research

Long-term effectiveness study: Carry out a long-term follow-up study to assess the long-term effects of BFRT on the indicators of muscle strength, balance function, and fall risk of the elderly, and analyze its relationship with quality of life, self-care ability, and social participation.

Comparative study: Compare and contrast BFRT with traditional resistance training and balance training methods, compare their advantages and disadvantages in preventing falls in the elderly, and explore the best training program.

Multi-center study: Carry out multi-center clinical trials to expand the sample size and improve the reliability of the research results, to provide stronger evidence for the promotion and application of BFRT.

Continued Attention to Safety Studies

Research on different populations: Study the safety of BFRT for different groups of elderly people, such as those with chronic diseases, osteoporosis, cardiovascular diseases, etc., and assess the risks and benefits.

Potential risk assessment: Focus on the potential risks that BFRT may bring, such as muscle damage, vascular damage, thrombosis, etc., and formulate corresponding preventive measures and coping strategies.

Standardization: Based on the results of the study, develop more standardized standards for BFRT, including taping pressure, training intensity, training frequency, training time, etc., to ensure the safety of training.

Technological Innovation and Application Promotion

Intelligent equipment: develop more convenient and safer intelligent BFRT equipment, such as integrated blood pressure monitoring, heart rate monitoring, and other functions, as well as automatic adjustment of strap pressure, etc., to improve the safety, effectiveness, and convenience of training.

Virtual reality technology: Combine virtual reality technology with BFRT to create a more immersive training environment, improve interest and participation in training, and enhance the adherence of the elderly to training.

Telemedicine application: Utilizing telemedicine technology to provide remote BFRT guidance for the elderly, solving the problems of lack of professionals and geographical limitations, and facilitating home training for the elderly.

## Conclusions

Falls in the elderly are caused by a combination of factors, and prevention and control of risk factors are effective measures to reduce falls. Based on the latest research results, BFRT shows great potential in preventing falls in the elderly, not only can it effectively improve the exercise and cardiorespiratory function of the elderly, but also can improve the cognitive function and perception of the elderly, effectively reducing the risk of falls, and at the same time, has a good safety profile. BFRT can also formulate personalized treatment plans, according to the physical conditions of the elderly, and set up suitable training programs, effectively guaranteeing the effectiveness of the training. It can effectively ensure the training effect. In the future, we need to continue to strengthen the research, improve the training specifications, and promote the application of BFRT, so that it can be more widely used in the prevention of falls in the elderly, and effectively improve the quality of life of the elderly.
